# Topological and spatial heterogeneity of gut microbiota co-abundance networks in pigs revealed by using large-scale samples

**DOI:** 10.3389/fmicb.2025.1578236

**Published:** 2025-06-25

**Authors:** Lin Wu, Yuxin Liu, Congying Chen, Jun Gao

**Affiliations:** National Key Laboratory of Pig Genetic Improvement and Germplasm Innovation, Jiangxi Agricultural University, Nanchang, China

**Keywords:** co-abundance network, gut microbiota, spatial heterogeneity, 16S rRNA gene, pigs

## Abstract

Fecal samples have often been used as a proxy for studying the gut microbiota. However, the fecal microbiota does not fully reflect the gut microbiota composition. To elucidate the biogeographical characteristics and interaction networks of porcine gut microbiota, we systematically determined the compositions and co-abundance networks of gut microbiota from small to large intestine using 2,955 microbial samples from ileum, cecum, and feces of F6 (715) and F7 (687) pigs which were slaughtered at the age of 240 days from an experimentally designed heterogeneous pig population by crossing eight divergent breeds using 16S rRNA gene sequencing. The gut microbial composition showed significant spatial heterogeneity. The diversity of the gut microbiota progressively increased along the intestinal tract. Significantly spatial heterogeneity was also observed in the co-abundance networks. The numbers of OTUs showing co-abundance correlations with other OTUs were increased from ileum to cecum and feces. We found that the stronger the co-abundance correlation, the higher the gut location specificity of the co-abundance relationships. Only 644 (0.2%) co-abundance relationships among OTUs existed in all three gut locations. *Prevotella* had the highest number of stable co-abundance relationships, followed by Bacteroidales, *Bacteroides*, *S24-7*, and Lachnospiraceae. Topological analysis found that the co-abundance network of OTUs in the ileum showed random network characteristics, while the co-abundance networks of OTUs in the cecum and feces showed the scale-free network characteristics in both pig populations. Compared with the co-abundance networks in the cecum and feces, the networks in the ileum had fewer nodes, but more edges, indicating that the ileum microbiota was a microbial ecosystem with a smaller number of microbial species, but closer interactions. However, the pairwise co-abundance correlations between OTUs were more independent in the cecum. The co-abundance network in the ileum had the lowest stability, but the highest vulnerability, while the co-abundance network in the cecum exhibited the highest stability, but low vulnerability. Finally, we characterized the gut location-specific microbial co-abundance relationships. Characterizing the different phylogenetic structures of gut microbiota in different intestinal biogeographic niches would help to explore the spatial heterogeneity of microbial physiological functions and to develop the strategy regulating gut microbiota targeting to specific gut locations.

## Introduction

The mammalian gut microbiota is comprised of hundreds of microbial species and plays important roles in host physiological activities (Kinross et al., 2011). It influences the host immune system, health, and metabolism (Nowland et al., 2019; LU et al., 2024; Yang et al., 2024; Zhou et al., 2025). In pigs, gut microbiota has been reported to influence host fat deposition, feed efficiency, piglet diarrhea, and even sow estrus return (Gresse et al., 2017; Yang et al., 2017; Chen et al., 2021; Liu M. et al., 2023). The composition and diversity of pig gut microbiota are affected by diets (Ma et al., 2022), growth stage, sex, breed, and gut location (Jensen, 1998; Upadhaya and Kim, 2022). Moreover, the microbial distribution is also influenced by the spatial organization of the intestine, which can be thought to distribute along two axes, a longitudinal axis (from mouth to rectum) and a radial axis (from mucosa to lumen) (Tropini et al., 2017). The prevalence and diversity of microbes in different locations of the gastrointestinal (GI) tract appear to be dependent on various factors, such as availability of nutrients, pH levels, oxygen concentrations, and bacterial cooperation (Hollister et al., 2014; Pereira and Berry, 2017; Hishida and Iwasa, 2021). There were significant differences in the microbial composition, diversity, and abundance from small intestine to cecum and feces. For example, *Clostridium* is enriched in the small intestine, while *Prevotella* is more abundant in the cecum (Yang et al., 2016). Gut microbiota plays different roles in different gut locations. In the small intestine, microbiota are mainly involved in the digestion and absorption of nutrients, while microbiota in the large intestine are mainly involved in the degradation of nutrients indigestible in the small intestine (Wang et al., 2020). Comparison analysis of the microbiota among different gut locations suggested that the fecal microbiota could not fully represent the microbial compositions of the whole intestine (Ahn et al., 2023), although fecal samples are relatively easy to be obtained. Therefore, revealing the taxonomic composition of microbial communities and the interactions among microbes in different gut locations are important for studying the potential roles of the gut microbiome in host physiology, health and phenotypes.

Numerous members of gut microbiota do not inhabit in the GI tract independently, but form highly complex ecological interaction networks (Faust and Raes, 2012). These interactions contribute to many aspects of host physiology (Turnbaugh et al., 2009; Thaiss et al., 2016). The complex interactions occurring among microbial taxa make the function of the collective microbiome more than the function of any of its constitute species. Moreover, the complexity characteristics of interaction structures within gut microbiota could be well modelled as networks (Layeghifard et al., 2017). Studying co-abundance microbial groups could capture the overall connectivity within the gut microbiome (Boetto et al., 2024) and better understand the interactions between individual microbes within the microbial community. Furthermore, constructing co-abundance networks could investigate the balance between bacteria species and identify abnormal ecological interactions (Chen et al., 2020; Liu Y. et al., 2023; Wu et al., 2024). Therefore, the biologically important clusters from a phylogenetic interaction network should provide critical insights into their structure, connectivity, function and overall organization. However, spatial heterogeneity of co-abundance networks of gut microbiota from small intestine to cecum and feces with large sample size from healthy pigs has largely unknown.

To systematically evaluate the spatial heterogeneity of the co-abundance networks of gut microbiota, in this study, we used 2,955 microbial samples including ileum content, cecum content, and feces samples from F6 and F7 pigs of a mosaic pig population. Microbial compositions of different gut locations were determined by 16S rRNA gene sequencing. We constructed a biogeography map of the pig gut microbial compositions in healthy pigs. And then, co-abundance networks were constructed to evaluate the spatial heterogeneity of interaction networks among microbes in both F6 and F7 pig populations. The topological characteristics of co-abundance networks of gut microbiota was also systematically described. This study provided an important insight about the spatial heterogeneity of pig gut microbial compositions and their interaction networks in healthy pigs, and also gave a reference for improving pig health by regulating gut microbiota.

## Materials and methods

### Animals

A total of 715 F6 and 687 F7 pigs from a mosaic population were used in this study. This mosaic population was constructed with eight founder pig breeds including four Western commercial pig breeds Duroc, Landrace, Large White, and Pietrain, and four Chinese indigenous pig breeds Bamaxiang, Erhualian, Laiwu, and Tibetan. All experimental pigs were raised in the same farm house under the uniform conditions and fed twice a day with commercial corn-soybean formula diets containing 16% crude protein, 3,100 kJ digestible energy, 0.78% lysine, 0.6% calcium, and 0.5% phosphorus (Supplementary Table S1). Water was available *ad libitum* from nipple drinkers. All boars were castrated at 80 days of age. The animals were healthy and had not received antibiotic treatment within 2 months before sample collection. All experimental pigs were slaughtered at 240 ± 3 days by bleeding after electrical stunning.

### Microbial sample collection and DNA extraction

Fecal samples from experimental pigs were collected from the rectum before pigs were transported to the slaughter house. Luminal content samples from the ileum and cecum were harvested within 30 min after slaughter. A total of 304 ileal contents, 308 cecum contents, and 691 fecal samples were obtained from F6 pigs. And 411 ileal contents, 651 cecum contents, and 590 fecal samples were harvested from F7 pigs.

All samples were placed in 2-ml sterile centrifuge tubes and dipped in liquid nitrogen immediately. After transported to the laboratory, all samples were stored at −80°C freezer until DNA extraction. Genomic DNA was extracted with the QIAamp DNA Stool Mini Kit (Qiagen, Germany) according to the manufacturer’s instructions. The quantity and quality of all isolated DNA samples were measured by agarose gel electrophoresis and a Nanodrop-1000 spectrophotometer (Nanodrop Technologies, United States).

### Amplification and sequencing of bacterial 16S rRNA gene

The V3–V4 hypervariable region of the 16S rRNA gene was amplified by PCR with the barcode fusion forward primer 338F [ACTCCTACGGGAGGCAGCAG] and the reverse primer 806R [GGACTACHVGGGTWTCTAAT] under the melting temperature of 55°C with 28 cycles. The sequencing of PCR amplicons was performed on a MiSeq platform (Illumina, United States) according to the manufacturer’s manual.

16S rRNA gene sequencing data were processed with the methods described in detailed in our previous report (Yang et al., 2022). In brief, raw 16S rRNA gene sequencing reads were demultiplexed. Primer and barcode sequences were trimmed using Trimmomatic (v.0.39) (Bolger et al., 2014). Reads with ≥10 consecutive identical or ambiguous bases were removed. Clean paired-end reads were merged (at least 10 bp overlap) into tags using FLASH (v.1.2.11) (Magoč and Salzberg, 2011). Chimeric reads were removed using USEARCH (v.7.0.1090) (Edgar, 2010). Tags were clustered into OTUs using VSEARCH (v.2.8.1) (Rognes et al., 2016) using the 97% sequence identity as a similarity threshold. The Greengenes (v.13.5) database and RDP classifier (v.2.2) (Wang et al., 2007) were used to match OTUs to taxa, and OTUs and taxa present in more than 10% of the samples with an abundance higher than 0.01% were retained.

### Construction of microbial co-abundance network

OTUs present in at least 10% of samples and having relative abundance of at least 0.01% were selected to construct co-abundance networks. Fastspar software (v1.0.0) based on the sparse correlation of component data (SparCC) algorithm was used to calculate the pairwise correlations among 279 OTUs in ileum content samples, 891 OTUs in cecum content samples, and 1,016 OTUs in feces samples in F6 pigs based on the OTU abundances. The numbers of OTUs used for constructing the co-abundance networks were 184, 1,022, and 1,120 in ileum, cecum, and feces samples of F7 pigs, respectively. The OTUs with statistically significant correlations (*p* < 0.05) were retained for subsequent analysis.

### Identification of co-abundance OTU pairs that are stably present in different gut locations

Co-abundance relationships among OTUs present in all three gut locations were analyzed in both F6 and F7 populations. Briefly, co-abundance relationships among OTUs were first analyzed in each gut location separately in the F6 and F7 populations using the Cochran-Q test with the metagen() function from the meta package (v8.0.1) in R, which used an inverse variance weighted method to calculate the squared differences of the effects between individual and combined studies. *p*-values of the Cochran-Q test were recorded for each co-abundance relationship across three gut locations, and co-abundance relationships of OTUs with *p* > 0.05 were considered stable among gut locations.

### Dividing the modules of co-abundance networks and analyzing their topological characteristics

Co-abundance relationships among OTUs with correlation coefficients greater than 0.5 were retained for constructing co-abundance networks in three different gut locations of F6 and F7 pig populations. The modules of co-abundance networks were delineated using the cluster_fast_greedy() function from the igraph package (v2.1.2) in R, and were further visualized using Cytoscape software (v3.10.2). The microeco package (v1.11.0) in R was used to calculate the topological properties (number of nodes, number of edges, average node degree, clustering coefficient, centrality, modularity) and node vulnerability of the co-abundance networks in three gut locations. The nodes were classified according to intra-module connections and inter-module connections. If the node belonged to a module hub (Zi > 2.5, Pi<0.62), connector (Zi > 2.5, Pi>0.62), or a network hub (Zi > 2.5, Pi>0.62), it was considered as a hub node of the network, and if the nodes were recognized as the peripheral nodes (Zi < 2.5, Pi<0.62), they were considered as non-hub nodes of the network.

The loss in network efficiency when the node and all of its edges were removed was treated as node vulnerability (Costa et al., 2007). Vulnerability reflected how much a node contributed to the global efficiency of the network. The maximum of node vulnerability indicated the vulnerability of the entire network. Low vulnerability indicated a more stable network (Kajihara and Hynson, 2024). Node Vulnerabilities in the co-abundance networks of three gut locations were calculated using the vulnerability () function in the microeco package (v1.11.0) in R.

### Assessment of the network stability

Before evaluating the stability of the co-abundance networks of OTUs in different gut locations of F6 and F7 populations, the strength of abundance-weighted mean interactions of nodes was calculated to evaluate the influence of each node in the network. Then, the nodes that simultaneously existed in the co-abundance networks in all three gut locations (referred to as the core nodes) were identified, and the network stability was determined based on the abundance-weighted mean interaction strength (wMISi) (Li et al., 2023), the number of core nodes, and the total number of nodes.

### Evaluating the robustness of co-abundance networks

Network robustness was defined as the ability of a network to resist node loss after random or targeted removal of nodes (Kajihara and Hynson, 2024; Li et al., 2024). The effect of hub bacteria on the robustness of the microbial co-abundance network was evaluated with the methods of hub and random non-hub bacteria removal. In both methods, 50% of bacteria were randomly removed, and the ratio of the remaining wMISi to the total network wMISi was calculated as the network robustness indicator. The simulation was repeated 10 times to ensure robustness of the results.

### Statistical analysis

The alpha-diversity of gut microbiota, including Chao1, ACE, observed species richness, Simpson, and Shannon indices were calculated using the vegan package (v2.6–8) in R. The abundances of bacterial taxa and the alpha-diversity indices between two groups were compared using the Wilcoxon *t*-test. *p* values were adjusted for multiple testing using the Benjamini-Hochberg (BH) method. The significance threshold was set at the false discovery rate (FDR) ≤ 0.05. Bray-Curtis distances were calculated using the vegdist() function of the vegan package (v2.6–8). PERMANOVA was performed by adonis2 in R vegan with 999 permutations. The *p* values were adjusted for the multiple tests with Benjamini–Hochberg. And the beta-diversity of microbial communities at different gut locations was compared using the Principal Coordinate Analysis (PCoA).

## Result

### Spatial heterogeneity of pig gut microbial compositions along the intestinal tract

A total of 1,303 microbial samples were collected from F6 pigs, including 304 ileum content samples, 308 cecum content samples, and 691 feces. The numbers of OTUs detected in ileum, cecum, and feces samples were 6,448, 9,876, and 11,291, respectively, containing a total of 5,679,424, 6,015,428, and 13,412,460 clean reads. After quality control based on the abundance and prevalence (See the Methods), 280, 891, and 1,016 OTUs were retained for further analysis in ileum, cecum and feces samples, respectively.

**Figure 1:**
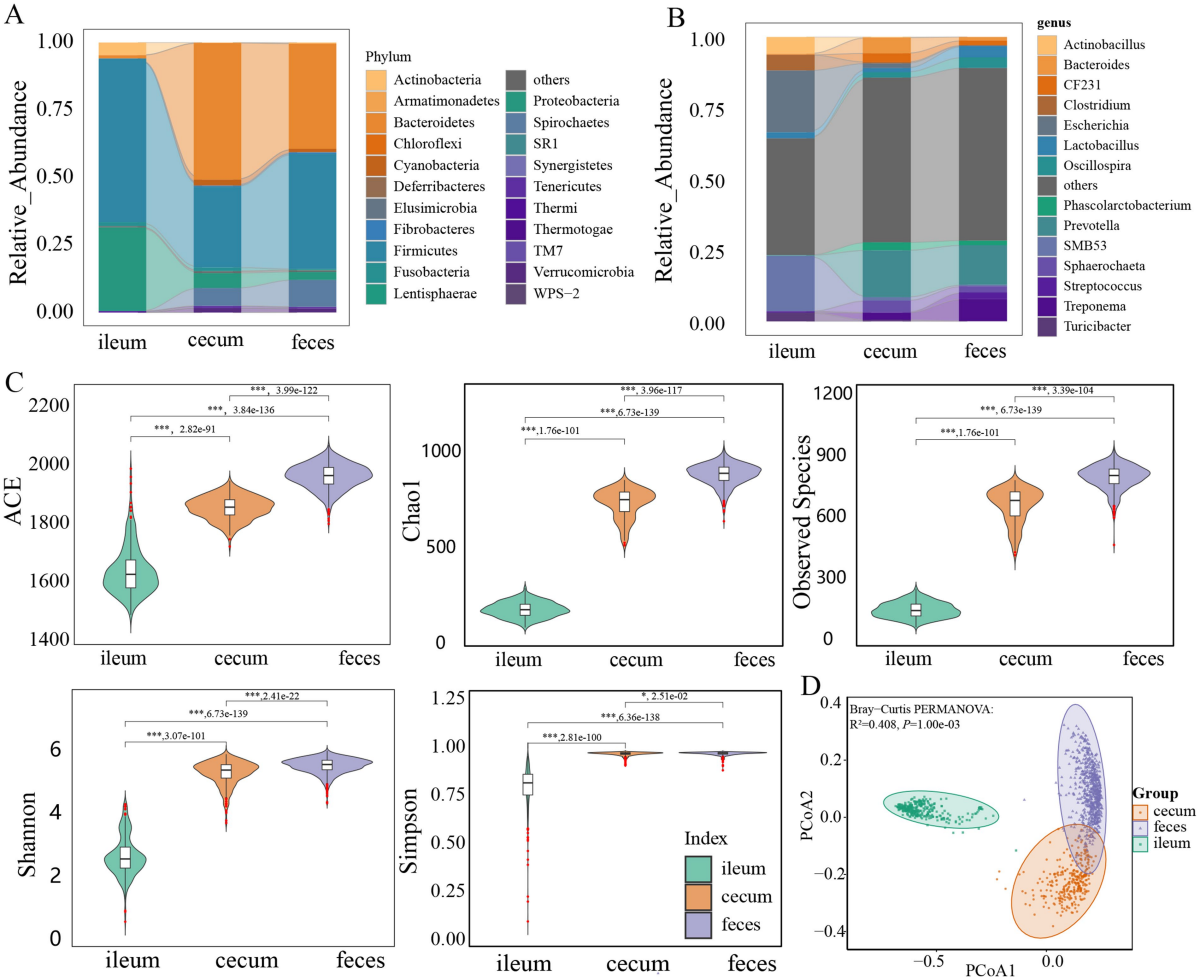
Comparison of the microbial composition and diversity among three gut locations. Comparison of the bacterial composition among ileum, cecum, and feces at the phylum **(A)** and genus level **(B)**. **(C)** Comparison of the alpha-diversity of microbial composition among ileum (*n* = 304), cecum (*n* = 308), and feces (*n* = 691). ACE, Chao1, observed species, Shannon, and Simpson indices were analyzed. **(D)** PCoA analysis of the microbial composition in ileum, cecum, and feces based on Bray-Curtis distance. All these results were obtained in F6 pigs. The results in F7 pigs are shown in Supplementary Figure S1. The comparison was performed using the Wilcoxon *t*-test. *p* values were adjusted for multiple testing using the Benjamini-Hochberg (BH) method. * *p* < 0.05, ** *p* < 0.01, *** *p* < 0.001.

The microbial compositions and relative abundances in the gut of F6 pigs changed significantly from the ileum to the cecum and feces. Firmicutes (60.83%) and Proteobacteria (31.17%) together accounted for approximately 92.00% of the total abundance of the gut microbiota in the ileum. However, the relative abundances of these two phyla decreased to 35.71 and 45.96% in the cecum and feces, respectively. Meanwhile, the relative abundance of Bacteroidetes increased significantly from 1.00% in the ileum to 50.96 and 39.16% in the cecum and feces, respectively. Firmicutes and Bacteroidetes together dominated the microbial composition in the cecum and feces, and accounted for more than 81.07 and 82.09% of total abundance, respectively (Figure 1A). At the genus level, *Escherichia*, *SMB53*, *Actinobacillus*, *Clostridium*, and *Lactobacillus* were the predominant bacterial genera in the ileum. Especially, *Escherichia* and *SMB53* occupied 41.27% of relative abundance, whereas *Prevotella*, *Bacteroides*, *Sphaerochaeta, CF231*, *Phascolarctobacterium*, and *Treponema* had high abundances in the cecum, and the relative abundances of *Prevotella*, *Treponema*, *Lactobacillus*, *Oscillospira*, *Streptococcus,* and *Sphaerochaeta* were listed in the top six in feces samples. In particular *Prevotella* had 16.49% of relative abundance in the cecum and 13.95% in feces samples (Figure 1B).

The similar microbial compositions were also observed in three gut locations of F7 pig population. Firmicutes had high abundance in all ileum (66.11%), cecum (24.73%), and feces samples (39.28%). Proteobacteria accounted for 26.72% of relative abundance in the ileum microbiota. Bacteroidetes showed 51.46 and 40.58% of relative abundances in the cecum and feces microbiota, respectively (Supplementary Figure S1A). At the genus level, the dominant bacterial genera in each of three gut locations were similar to that in the F6 population. *Escherichia* and *SMB53* together occupied 55.56% of total abundance in the ileum microbiota. *Prevotella* had 12.88 and 11.61% of relative abundances in the cecum and feces microbiota, respectively (Supplementary Figure S1B).

The observed species, Chao 1, ACE, Shannon, and Simpson indices (alpha-diversity indices of gut microbiota) showed significant differences among three gut locations (*p* < 0.05). Compared with that in the ileum, these indices in the cecum and feces samples increased significantly (Figure 1C), suggesting that the diversity of the gut microbiota progressively increased along the gut tract from ileum to cecum and feces. Beta-diversity was assessed by the PCoA based on the Bray-Curtis distance. It was clear that the microbiota composition of ileum samples was distinctly different from the microbial compositions of cecum or feces samples (*R^2^* = 0.408, *p* = 0.001) (Figure 1D). The similar results were also observed in the F7 population. Both alpha- and beta-diversity of microbial compositions were significantly different among three gut locations (Supplementary Figures S1C,D).

### Construction of the co-abundance networks of microbial OTUs in three gut locations of F6 and F7 pig populations

**Figure 2:**
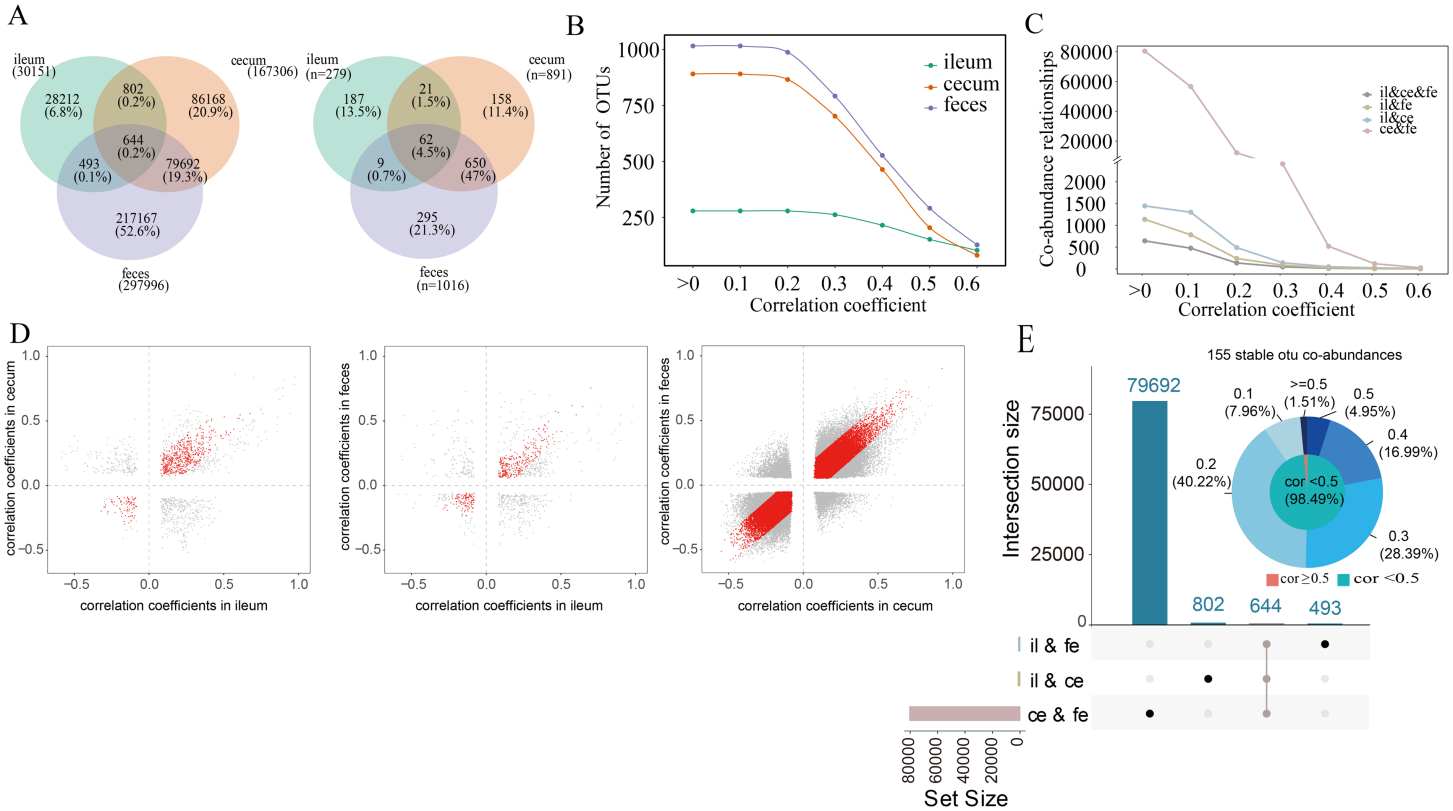
Co-abundance relationships among OTUs detected in each of three gut locations. **(A)** The number of co-abundance relationships identified in the ileum, cecum and feces of the F6 pig population (left) and the number of OTUs involved in these co-abundance relationships (right); **(B)** The changes in the numbers of co-abundance relationships identified in the ileum, cecum and feces of the F6 populations following the increase of correlation coefficients. The *X*-axis represents correlation coefficients, and the *Y*-axis indicated the number of OTUs with co-abundance relationships. **(C)** The number of co-abundance relationships shared among different gut locations following the increase of correlation coefficients in the F6 populations. The *X*-axis represents correlation coefficients, and the *Y*-axis indicates the number of OTU co-abundance relationships. **(D)** Evaluating the stability of co-abundance relationships between two gut locations in the F6 population. Each point represents a co-abundance relationship among OTUs. The horizontal and vertical axes represent the correlation coefficients of co-abundance relationships. Red dots indicate co-abundance relationships with no significant difference in effect size between two gut locations with *p* > 0.05 in Cochran’s Q test. **(E)** The numbers of co-abundance relationships shared between two gut locations and among all three gut locations (boxplot) and the distribution of the correlation coefficients for 155 stable co-abundance relationships (pie chart). The values on the outside circle of the pie chart represent different correlation coefficients, and the values in brackets are the ratios of stable co-abundance relationships in 155 stable co-abundance relationships in each correlation coefficient interval. The results in F7 pigs are shown in Supplementary Figure S2.

The SparCC algorithm was used to predict sparse correlations of OTUs in the ileum, cecum, and feces samples. The microbial co-abundance networks in each of three gut locations demonstrated the high connectivity among OTUs, especially in the cecum and feces. In F6 pigs, significantly fewer number of co-abundance relationships were found in ileum samples compared to that in cecum and feces samples (Figure 2A). A total of 30,151 co-abundance relationships were identified among 279 OTUs in the ileum, 167,306 co-abundance relationships among 891 OTUs in the cecum, and 297,996 co-abundance relationships among 1,016 OTUs in the feces. Most of these co-abundance relationships were gut location-specific (Figure 2A). In more details, significantly spatial heterogeneity existed for the co-abundance networks in three gut locations. The numbers of OTUs showing co-abundance correlations with other OTUs were increased from ileum to cecum and feces under near all correlation coefficients (*r*) in F6 pigs although the numbers of OTUs detected correlations were decreased following the increase of correlation coefficient values in all three gut locations (Figure 2B). Furthermore, the numbers of shared co-abundance relationships between gut locations gradually decreased following the increased correlation coefficients, indicating that the stronger the co-abundance correlation, the higher the gut location specificity of the correlations (Figure 2C). There were significantly more shared co-abundance relationships between cecum and feces samples, followed by the number of shared co-abundance relationships between ileum and cecum (Figure 2C). Similar results were also observed in the F7 pig population. However, different from that in F6 pigs, the numbers of OTUs showing co-abundance correlations with other OTUs were similar between cecum and feces samples when the correlation coefficient was >0.4 (Supplementary Figure S2B,C).

In the microbial co-abundance networks of different gut locations, only 644 (0.2%) co-abundance relationships among OTUs existed in all three gut locations (Figure 2E). Among them, 155 relationships (24.07%) showed no significant difference among ileum, cecum and feces in the Cochran-Q test (*p* > 0.05), and were considered as the stable correlations across gut locations in the F6 population. Most of these 155 stable co-abundance relationships (98.49%) had the correlation coefficient less than 0.5 (Figure 2E). A total of 80,336 (79,692 + 644) co-abundance relationships were identified in both cecum and feces samples, among which, 60,902 relationships (75.81%) were stable in both gut locations. There were 1,446 co-abundance relationships among OTUs that were shared between ileum and cecum, of which 645 (44.61%) co-abundance relationships were stable in both ileum and cecum. And there were 1,137 co-abundance relationships shared in both ileum and feces, of which 425 (37.38%) co-abundance relationships were stable in both gut locations (Figure 2D). The percentage of stable co-abundance relationships between cecum and feces (75.81%) was significantly higher than that between ileum and cecum, between ileum and feces, and across all three locations (Figure 2D).

**Figure 3:**
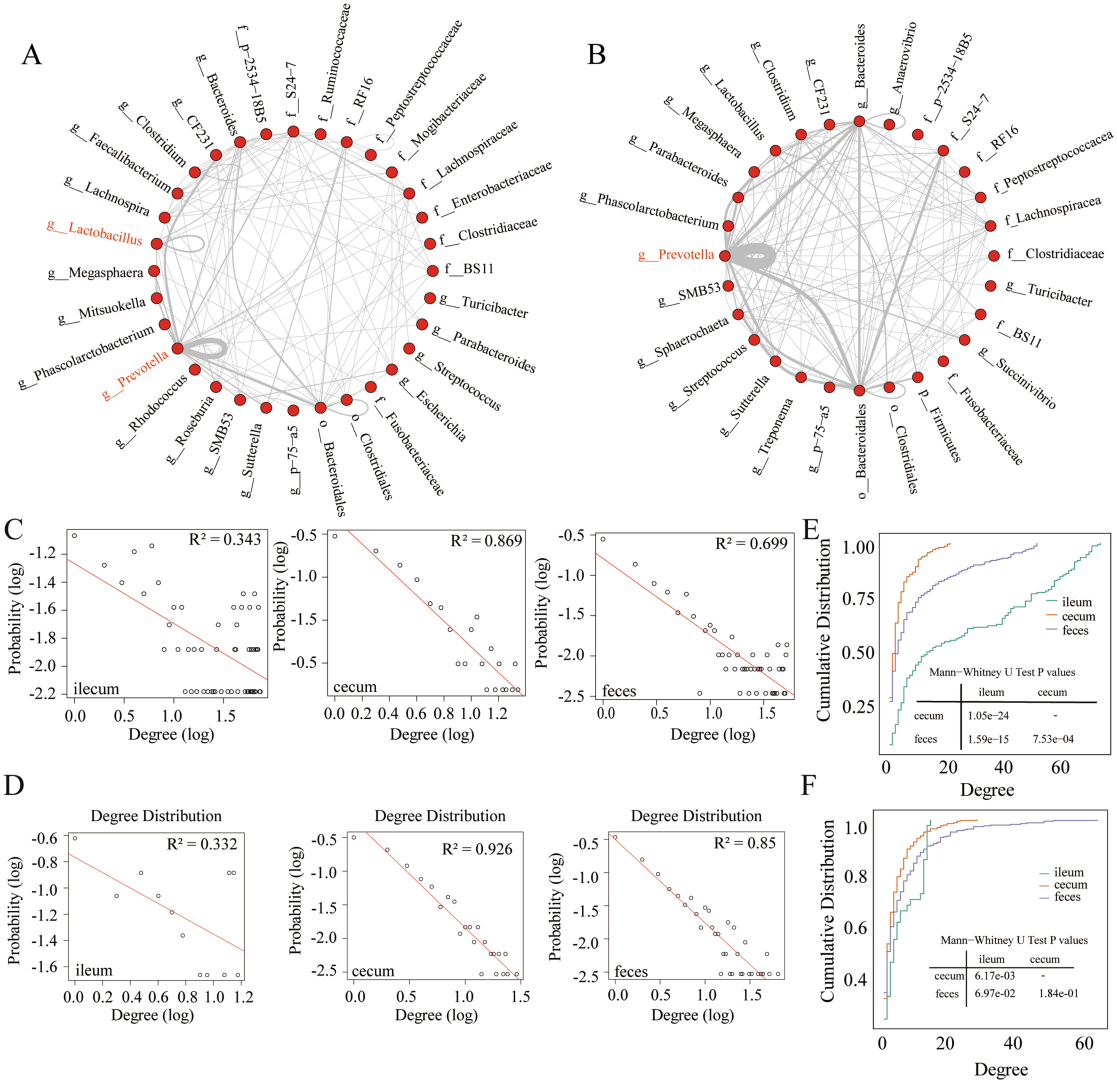
Interaction characteristics and node degree of stable co-abundance relationships existing in all three gut locations. **(A)** The interaction characteristics of 155 stable co-abundance relationships existing in all three gut locations of the F6 pig populations. **(B)** The interaction characteristics of 247 stable co-abundance relationships existing in all three gut locations of F7 pigs. Each point represents a bacterial taxonomy. Each line represents a co-abundance relationship. **(C,D)** Node degree of co-abundance networks in the ileum, cecum, and feces of the F6 population **(C)** and F7 population **(D)**. The *X*-axis represents the logarithmic node degree, and the *Y*-axis shows the logarithmic node degree probability. The red straight line indicates the fitting curve of the node degree distribution. **(E,F)** Node degree cumulative distribution curves of co-abundance networks in the ileum, cecum, and feces of the F6 population **(E)** and F7 populations **(F)**. The *X*-axis represents the node degree, and the *Y*-axis represents the cumulative distribution.

There were 678,411 co-abundance relationships constructed by 2,326 OTUs in the F7 population. Among them, 861 co-abundance relationships existed in all three gut locations (Supplementary Figure S2E). A total of 247 (33.45%) co-abundance relationships were stable in the Cochran-Q test (Supplementary Figure S2E). The numbers of gut location-specific OTUs with co-abundance relationships were 85, 357, and 471 for ileum, cecum, and feces samples, respectively (Supplementary Figure S2A). Consistent with the results in the F6 population, the percentages of stable co-abundance relationships between ileum and cecum (44.69%), and between ileum and feces (43.84%) was significantly lower than that between cecum and feces (65.42%) (Supplementary Figure S2D). Overall, the distribution of stable and gut location-specific microbial co-abundance relationships indicated the significant differences in the interaction networks of gut microbiota between ileum and large intestine (cecum and feces). Additionally, strongly microbial interactions were more likely gut location-specific.

### Interaction characteristics of stable microbial co-abundance relationships in different gut locations at the taxonomic level

Taxonomic annotations were performed for OTUs with stable co-abundance relationships that were observed in all three gut locations of F6 (155 relationships) and F7 pig populations (247 relationships). As shown in Figures 3A,B, stable co-abundance relationships were predominantly observed in several bacterial taxa in both pig populations. *Prevotella* exhibited the highest number of stable co-abundance relationships, followed by Bacteroidales, *Bacteroides*, *S24-7*, and Lachnospiraceae. In F6 pigs, we identified stable co-abundance relationships among OTUs within the same genus, such as the OTUs in *Prevotella*, *Lactobacillus*, and *Clostridium*, especially in *Prevotella* (Figure 3A). In F7 pigs, the stable co-abundance relationships among OTUs within the same genus were mainly found in *Prevotella*. The high numbers of stable co-abundance relationships were identified among OTUs across different taxa. For examples, the OTUs within *Prevotella* were co-abundantly correlated with the OTUs within Bacteroidales, *Bacteroides*, and *CF231* (only in F6 pigs). And the OTUs within Bacteroidales showed stable co-abundance relationships with the OTUs within *Bacteroides* and *S24-7* (Figure 3B).

### Topological characterization of co-abundance networks in different gut locations

To analyze the topological structures of co-abundance networks in each of three gut locations and identify the gut location-specific networks, we retained those microbial co-abundance relationships with correlation coefficients greater than 0.5 (robust correlations) in the F6 and F7 populations. First, we analyzed the node degree of co-abundance networks in three gut locations of the F6 and F7 pig populations. From the probability distribution and the cumulative distribution of node degrees, we found that the co-abundance network of OTUs in the ileum showed random network characteristics, while the co-abundance networks of OTUs in the cecum and feces exhibited the scale-free network characteristics in both populations (Figures 3C–F). This result indicated that the microbial co-abundance networks gradually evolved from random networks to scale-free networks from ileum to cecum and feces.

**Figure 4:**
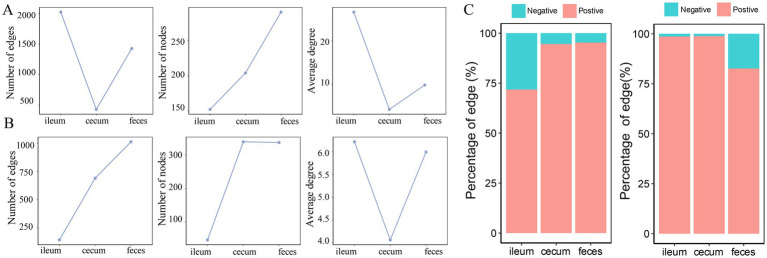
Comparison of the numbers of nodes, edges and average node degree of the co-abundance networks in different gut locations. Topological structural characteristics of co-abundance networks in the ileum, cecum, and feces of the F6 population **(A)** and F7 population **(B)**, including the number of nodes, the number of edges, and average node degrees in different gut location. **(C)** The percentages of positive and negative interactions in in the co-abundance networks of ileum, cecum, and feces in the F6 population and the F7 population.

In the F6 population, compared with the co-abundance networks in the cecum and feces, the microbial co-abundance network in the ileum had fewer nodes, but more edges, indicating a “streamlined and efficient” microbial ecosystem with few microbial species but closer interactions in the ileum microbiota. The microbial co-abundance network in the cecum had the fewest edges, and the pairwise co-abundance correlations between OTUs were more independent, suggesting the relatively independent ecological functions for microbes in the cecum (Figure 4A). In the F7 population, the co-abundance network of the ileum was relatively simple, with fewer nodes and edges (Figure 4B). In contrast, the co-abundance networks of the cecum and feces were more complex. However, the distribution of the average node degrees was consistent with the result in F6 pigs. In addition, positive correlations were significantly more than negative correlations in all co-abundance networks of three gut locations in both pig populations. The number of negative correlations was considerably higher in feces samples than in the other two gut locations (Figure 4C). This result indicated that the interaction mode between these bacteria was dominated by synergy.

### Clustering coefficient, centrality, and modularity of co-abundance networks in different gut locations

**Figure 5:**
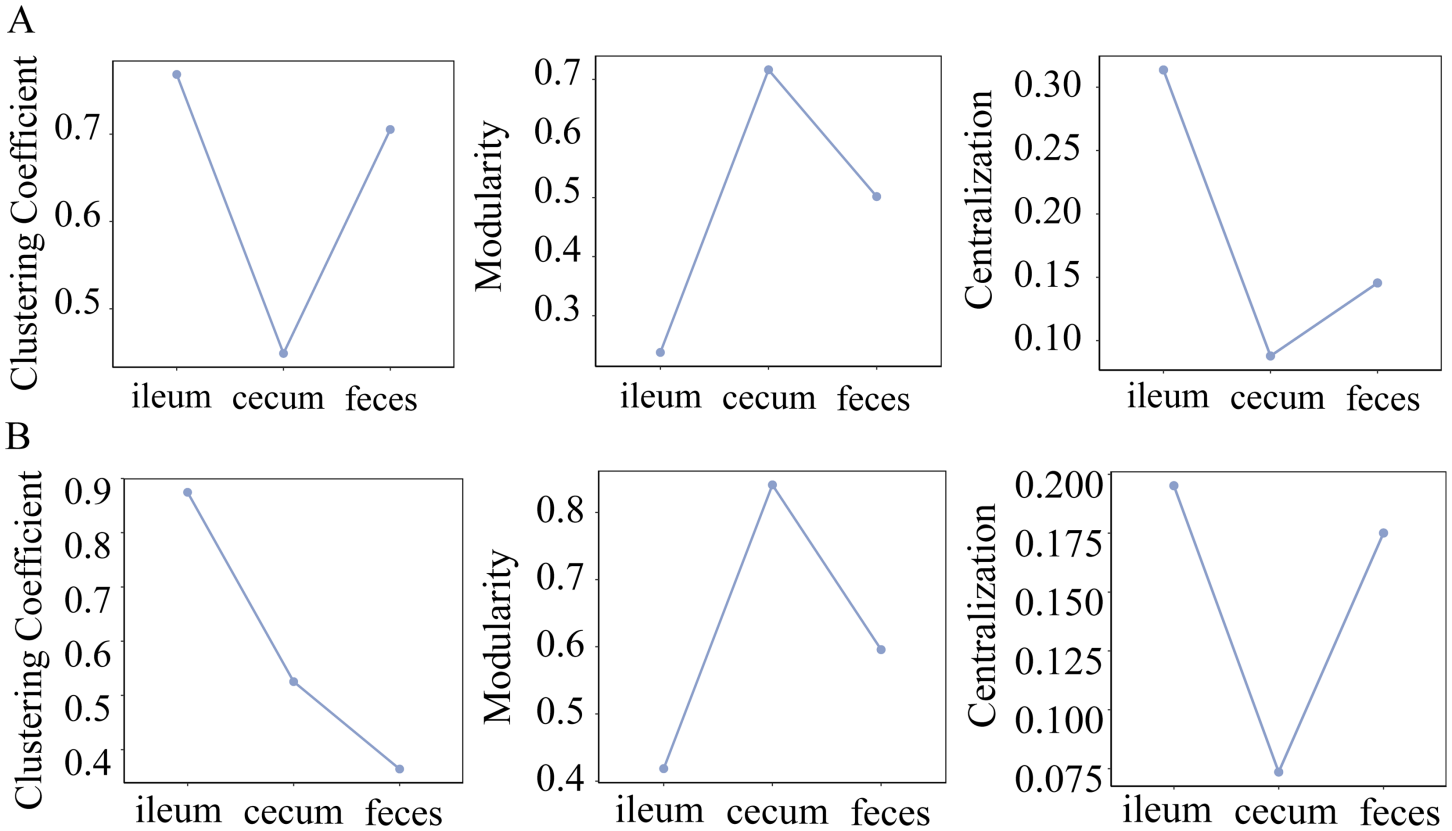
Comparison of clustering coefficient, modularity and centrality of the co-abundance networks in different gut locations. The topological characteristics of co-abundance networks constructed in the ileum, cecum, and feces of the F6 **(A)** and F7 populations **(B)**, including the indices of clustering coefficient, modularity, and centralization.

The clustering coefficient measures the degree of connection between the network nodes, and centralization has been used to measure whether there are a few central nodes with high connectivity in the network. In both F6 and F7 populations, the microbial co-abundance network in the ileum had the highest clustering coefficient and centralization, but exhibited the lowest modularity. However, the co-abundance network in the cecum showed the opposite tendency although it was strange that feces samples had the lowest clustering coefficient in F7 pigs (Figure 5). This result suggested that members in the microbial community of the ileum were closely connected with high synergy and integrality, while bacteria in the microbial community of the cecum were loosely connected and contained multiple independent functional modules that played roles independently.

**Figure 6:**
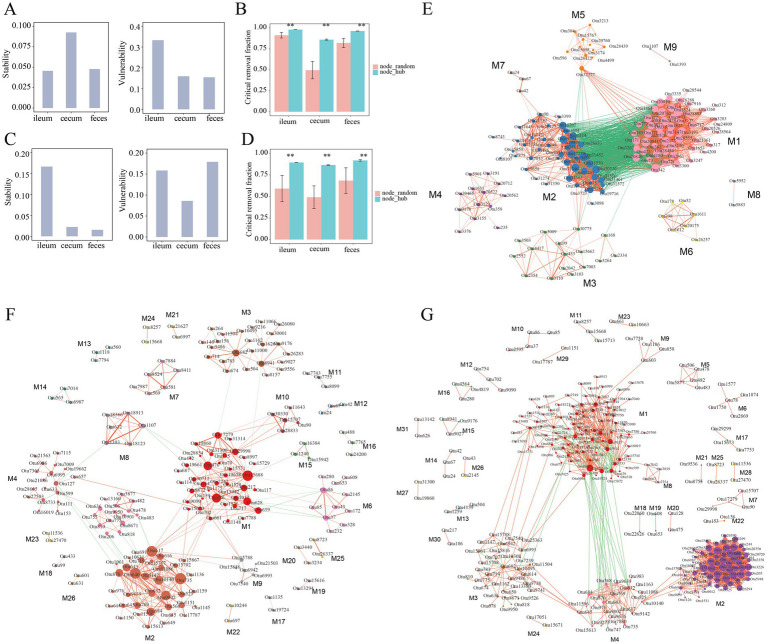
Stability, vulnerability, robustness, and modules of the co-abundance networks in different gut locations. **(A)** The stability and vulnerability of co-abundance networks constructed in the ileum, cecum, and feces of the F6 population. **(B)** Robustness evaluation of co-abundance networks in three gut locations of the F6 population. **(C)** The stability and vulnerability of co-abundance networks in the F7 population. **(D)** Robustness evaluation of co-abundance networks in three gut locations of the F7 population. Different evaluation strategies are represented by different colors. node_random: random removal of non-hub bacteria, node_hub: target removal of hub bacteria. **p < 0.01 after corrected the multiple tests. **(E–G)** Modules of co-abundance networks constructed in the ileum, cecum and feces samples in the F6 population. The correlations with coefficients > 0.5 were used to constructed the co-abundance networks. The numbers of modules in the networks of ileum, cecum and feces were 9, 26, and 31, respectively. Different colors meant different modules, the node size represents the degree of OTUs, red lines mean positive correlations and green lines indicate negative correlations. The thickness of the line between nodes shows the weight of the correlation coefficient between OTUs.

Next, the stability and vulnerability of the microbial co-abundance network in different gut locations were evaluated. In the F6 population, the co-abundance network in the ileum had the lowest stability, but the highest vulnerability, while the co-abundance network in the cecum exhibited the highest stability, but low vulnerability (Figure 6A). This result suggested that the ileum microbial community was easily influenced by gut environments, such as food changes, pH, and other factors, while the microbial community in the cecum was relatively stable. Compared to that in the cecum, the microbial co-abundance network in feces samples had low values of both stability and vulnerability. As we have known, the bacterial community of feces samples was easily affected by external environmental conditions. However, in the F7 population, the stability of the co-abundance networks in the cecum and feces was much lower than that in the ileum (Figure 6C). This could be attributed to the reason that the total numbers of nodes in the co-abundance networks of F7 pigs were significantly lower than that in F6 pigs. The effect of hub bacteria on the robustness of the microbial co-abundance networks was evaluated based on the hub bacterial removal method and the random removal method of non-hub bacteria (See methods). The result indicated that, compared to that in the random removal analysis of non-hub bacteria, the critical removal fractions of nodes for the disintegration of networks in three gut locations were significantly higher in the hub bacterial removal analysis indicating that hub bacteria played a key role in the robustness of the network (Figures 6B,D).

The co-abundance networks constructed in ileum, cecum, and feces samples of the F6 population could be divided into 9, 26, and 31 modules, respectively. Most of the OTUs in the same modules were positively correlated with each other. The module 1 in the co-abundance network of ileum content samples contained 52 OTUs which were mainly annotated to SCFA-producing bacteria, such as *Lactobacillus*, *Bacillus* and *Leuconostoc*. This module was negatively correlated with the module 2 in which 46 OTUs were mainly annotated to *SMB53*, *Clostridium*, *Epulopiscium*, and *Turicibacter*, but showed a positive correlation with the module 5 (Figure 6E). In the co-abundance network of cecum content samples, a total of 26 modules were obtained. As described above, OTUs in the same modules were positively correlated with each other, suggesting that OTUs in the same module might synergistically interact with each other. Among the top five modules with the highest number of OTUs, there was a negative correlation between module 2 and module 5. Most of the OTUs in the module 2 (36 OTUs) and module 7 (6 OTUs) were annotated to *Prevotella*. The Module 5 was mainly composed of OTUs belonging to *Bacteroides* and *Sphaerochaeta* (Figure 6F). The co-abundance network constructed in feces samples was comprised of 31 modules. The module 4 contained 24 OTUs belonging to *Prevotella* and showed positive correlations with each other. In addition, the module 4 showed a significantly positive correlation with the OTUs annotated to *Faecalibacterium prausnitzii* and *Prevotella copri* in the module 2 (65 OTUs) as well as with the OTUs annotated to *Prevotella* in the module 3 (33 OTUs) (Figure 6G; Supplementary Table S2).

The co-abundance networks constructed in ileum, cecum, and feces samples of the F7 population were comprised of 6, 38, and 37 modules, respectively. Consistent with the findings in the F6 population, most OTUs within the same module primarily exhibited positive interactions. Among six modules in the co-abundance network of ileum, except for module 3 and module 4, each module independently occupied a unique ecological niche. The module 1 contained the highest number of OTUs (20 OTUs), mainly consisting of OTUs from Clostridiaceae. The Module 2 was comprised of 10 OTUs that were mainly annotated to Clostridiaceae, *Epulopiscium*, and *Turicibacter*. All four OTUs in the module 4 belonged to *Lactobacillus*. A positive correlation was observed between *Turicibacter* (OTU42) in the Module 3 and *Lactobacillus* (OTU90) in Module 4. The co-abundance network of cecum content samples consisted of 38 modules with relatively simple interactions between different modules. The Module 1 contained 44 OTUs which were mainly annotated to *Bacteroides* and *Parabacteroides*. All OTUs in the Module 9 (13 OTUs) were derived from *Parabacteroides*, while the majority of OTUs in the Module 6 (22 OTUs) belonged to *Prevotella*. A positive correlation was observed between Module 1 and Module 9. In contrast, there was a negative correlation between *Bacteroides* OTUs in the Module 1 and *Prevotella* (OTU643) in the Module 6. The co-abundance network constructed in feces samples was comprised of 37 modules. The Module 1 contained 66 OTUs mainly annotated to SCFA-producing bacteria, such as *Prevotella*, *Coprococcus*, and *Faecalibacterium prausnitzii*. It exhibited complex interactions among module 2 (61 OTUs), module 3 (55 OTUs), and module 4 (22 OTUs). A total of 21 OTUs were included in the Module 5 and were mainly annotated to Treponema (Supplementary Figure S3; Supplementary Table S3).

### Gut location-specific microbial co-abundance relationships

**Figure 7:**
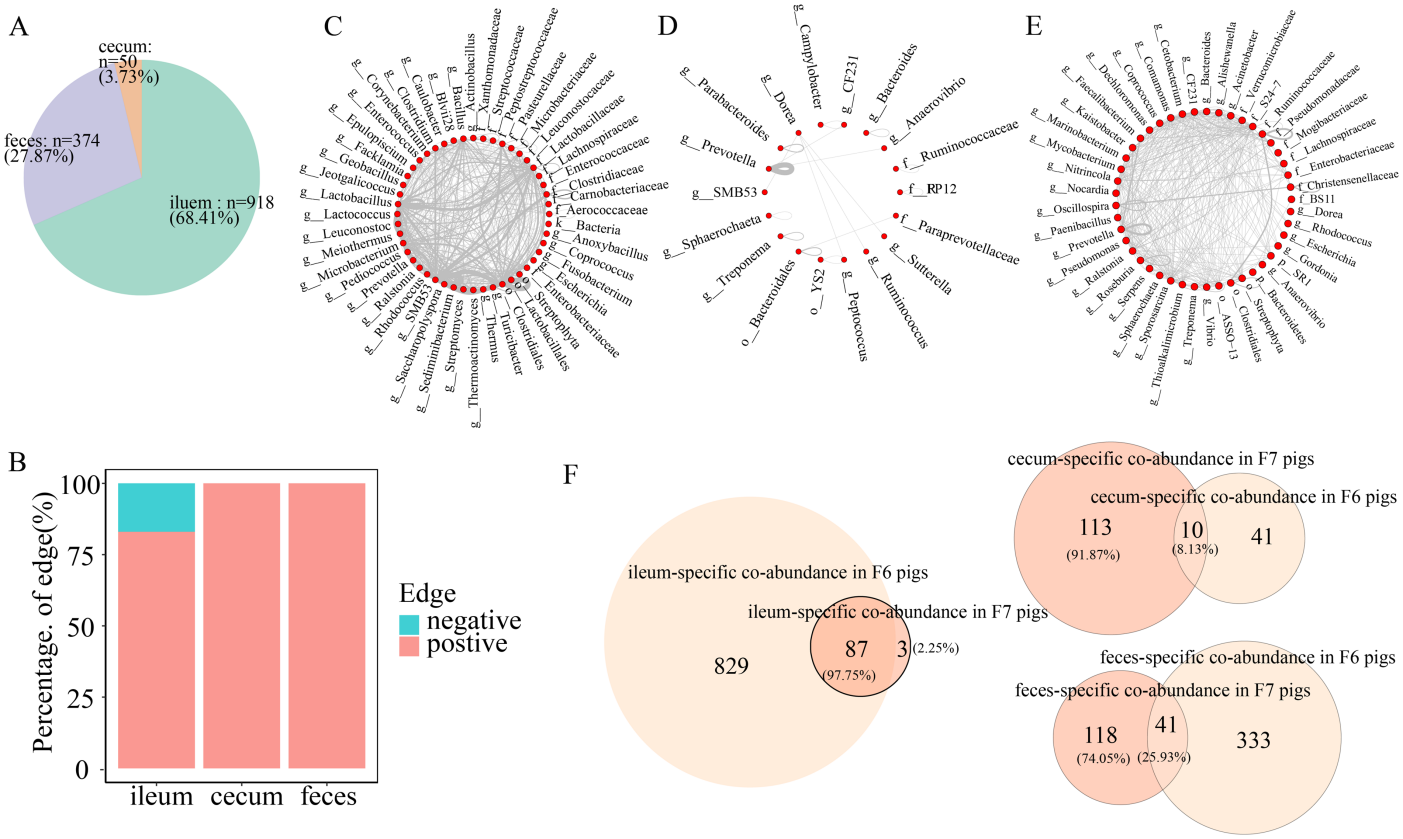
Gut location-specific co-abundance relationships in F6 pigs. **(A)** The proportion of gut location-specific co-abundance relationships identified in three gut locations of the F6 population. **(B)** The proportion of gut location-specific positive and negative relationships in the co-abundance networks of the ileum, cecum, and feces of the F6 population. **(C–E)** Co-abundance relationships specifically identified in the ileum **(C)**, cecum **(D)**, and feces **(E)** of F6 population at the taxonomic level. Each dot represents a microbial taxonomy. Each line represents a co-abundance relationship from the same or different taxa. The number of lines represents the number of co-abundance relationships identified in three gut locations. **(F)** The numbers and proportions of gut location-specific co-abundance relationships overlapped in the F6 and F7 populations.

Co-abundance relationships among OTUs were considered as the gut location-specific relationships if they satisfied one of the following criteria: (1) the difference in correlations between gut locations >0.6; or (2) the co-abundance relationships only existed in one gut location with a correlation coefficient ≥0.6 (She et al., 2024). Gut location-specific co-abundance relationships were found in all three gut locations in the F6 and F7 populations. In more detail, we identified 918 (68.41%), 50 (3.73%), and 374 (27.87%) gut location-specific co-abundance relationships in the ileum, cecum, and feces of F6 pigs, respectively (Figure 7A). The principal manifestation of the co-abundance relationships specifically identified in ileum samples was the existence of a large number of mutually exclusive relationships (negative correlations) (Figure 7B). These ileum-specific co-abundance relationships mainly occurred among OTUs within the same taxa, such as the OTUs within *Lactobacillus* and Clostridiales, and OTUs across different taxa (Figure 7C). We identified a significantly smaller number of the cecum-specific co-abundance relationships. These cecum-specific co-abundance relationships were positively correlated and mainly identified among OTUs annotated to the same genus (50% of the relationships were identified among the OTUs annotated to *Prevotella*) (Figure 7D). The co-abundance relationships specifically identified in feces samples only included positive correlations and occurred among OTUs within the same taxa (*Prevotella, Oscillospira,* and Ruminococcaceae) and OTUs across different taxa (Figure 7E).

In the F7 pig population, 89 (24.05%), 123 (33.24%), and 158 (42.71%) gut location-specific co-abundance relationships were identified in the ileum, cecum, and feces samples, respectively (Supplementary Figure S4A). These gut location-specific co-abundance relationships showed different patterns from those in F6 pigs. For examples, negative correlations were identified in feces samples. However, all correlations in ileum and cecum samples were positive (Supplementary Figure S4B). The ileum-specific co-abundance relationships mainly occurred among OTUs within *SMB53* and Clostridiales. A large number of the ileum-specific co-abundance relationships were also identified among SMB53-, *Turicibacter*-, and Clostridiales-OTUs (Supplementary Figure S4C). Moreover, different from that in the F6 population, 39.84% of the cecum-specific co-abundance relationships in the F7 population were observed among OTUs within *Bacteroides* (Supplementary Figure S4D). A significantly smaller number of feces-specific co-abundance relationships were identified in F7 pigs compared to F6 pigs. And most feces-specific co-abundance relationships were identified among OTUs within the same taxa, such as *Prevotella*, Ruminococcaceae, and *Treponema* (Supplementary Figure S4E). However, there were also small number of gut location-specific co-abundance relationships that were identified in both F6 and F7 pigs (Figure 7F).

## Discussion

A large number of studies have highlighted the differences in microbial compositions in different gut locations of pigs (Zhao et al., 2015; Crespo-Piazuelo et al., 2018; Peng et al., 2021). However, to our knowledge, most of these studies have only focused on the core microbiota and the gut microbial co-abundance networks were constructed in feces samples with small sample sizes. Few studies focused on the composition and co-abundance networks of the gut microbiota in different ecological niches of healthy experimental pigs. In this study, we used the large scale of 2,955 microbial samples from three gut locations of F6 and F7 populations of the mosaic pig families and systematically investigated the spatial heterogeneity of both gut microbial compositions and co-abundance networks of healthy pigs. Our results uncovered the significant spatial heterogeneity of pig gut microbial compositions and co-abundance networks. Especially, we characterized the topological characteristics of co-abundance networks in different gut locations, analyzed the stable co-abundance relationships across gut locations, and identified gut location-specific co-abundance relationships. The results suggested that the microbial composition information obtained from fecal samples alone could not represent the gut microbial structure. Revealing the gut location-specific differences of microbial compositions might provide accurate regulation targets for improving pig health and production performance.

Consistent with the results from previous studies (Isaacson and Kim, 2012; Looft et al., 2014; Song et al., 2022), the microbiota in the ileum was dominated by Firmicutes and Proteobacteria (Pollock et al., 2021), while the cecum and feces microbiota had high abundances of Bacteroidetes and Firmicutes (Shao et al., 2021; Rahman et al., 2024; Li et al., 2025). In both F6 and F7 populations, the gut microbial community showed a tendency of increased the alpha-diversity from the ileum to the cecum and feces. The small intestinal environment was harsh for the colonization and growth of some microbes (low pH, fast flow rate, and short residence time) (Barmpatsalou et al., 2021), which resulted in the low diversity of microbial composition (Possemiers et al., 2004; Donaldson et al., 2016). Different compositions of microbial communities in different gut locations further suggested that it was far from sufficient to study the gut microbiome by only using fecal samples.

Gut microbiota is a complex ecological system. Comprehensive interactions existed among microbes. There were close relationships in abundance between symbiotic or competing microbes. So, the construction of co-abundance networks should help to understand the interactions among microbes (Cao et al., 2022). We found that the number of co-abundance correlations among microbes significantly increased from ileum to cecum and feces. This was concordance with the increased complexity of gut microbial compositions from ileum to feces. However, the correlation coefficients for most of the co-abundance relationships were < 0.5, even for those co-abundance relationships that stably existed in all three gut locations. Spatial heterogeneity was found for co-abundance networks in different gut locations. Only 0.2% of co-abundance relationships were detected in all three gut locations. This should be caused by (1) different microbial compositions among three gut locations; (2) different gut environments, such as pH values, oxygen content, flow rate, and so on, which affected the interactions among microbial taxa. The gut microbial community is a complex ecosystem where microbial interactions are regulated by a variety of factors, and there may be many indirect or redundant effects in the co-abundance network, resulting in a single two-by-two linear relationship of low strength (Berry and Widder, 2014).

Taxonomic annotation found that many stable co-abundance relationships were identified on the OTUs belonging to the same genus. There were a large number of stable co-abundance relationships that occurred within the members of *Prevotella* or between the members of *Prevotella* and other taxa. This was consistent with the previous report that *Prevotella* formed functional groups with other common microbes, such as Bacteroides in the pig gut, and was the hub microbes of the microbial interaction network in the gut (Zhang et al., 2018). *Prevotella* is a key genus known to be abundant in the guts of pigs (Amat et al., 2020; Luo et al., 2022), *Prevotella* species can significantly increase fat deposition in pigs (Chen et al., 2021), and can serve as a potential biomarker for the levels of beneficial short-chain fatty acids (Sebastià et al., 2023). *Prevotella* colonizes in the gastrointestinal tract of a wide range of animals, makes important contributions to carbohydrate, lipid and amino acid metabolism (Jiang et al., 2021), and is significantly correlated with growth phenotype and feed efficiency in pigs (Kou et al., 2024).

The co-abundance relationships identified in all three gut locations were mainly synergistic (positive correlations) although they showed different topological characteristics that should reflect different ecological strategy. The network structure of the ileum microbiota was streamlined with high integrality and low number of network modules compared to that of cecum and feces microbiota. It exhibited higher clustering coefficients and centrality, indicating a more cooperative and integrated community structure. The small intestine is the key gut location for the digestion and absorption of most nutrients (Salminen et al., 1998; Jackson and McLaughlin, 2009; Wijtten et al., 2011). These topological characteristics might reflect close ecological interactions among microbial taxa in the ileum, which potentially support their roles in nutrient absorption, thereby contributing to the maintenance of local ecological stability. Moreover, microbes in the ileum should be more tightly interacted with each other to help fight against the hostile environment. The cecum is involved in the fermentation of carbohydrate (Mosenthin, 1998; Williams et al., 2001) and is a complex “fermentation tank” where microbes participate in the fermentation of cellulose and other indigestible dietary fiber (Varel, 1987). The co-abundance network of cecum microbiota displayed higher modularity and lower clustering coefficients, indicating a more scattered and functionally modular community structure. This structural characterization is often associated with greater ecological redundancy and systemic resilience, which might facilitate the preservation of microbial functions under host physiological fluctuations or environmental disturbances. As a metabolically active gut location, the microbial co-abundance network in the cecum microbiota had fewer connections, suggesting the microbial division of labor across diverse ecological niches and more independent roles of microbes in the cecum microbiota.

A large number of the gut location-specific co-abundance relationships were observed among OTUs belonging to the same taxa in all three gut locations of both F6 and F7 pig populations. This suggested that the bacterial species (strains) from the same genus (species) should promote the colonization and growth of each other. Unlike the cecum-specific co-abundance relationships, most of which were related to *Prevotella* or *Bacteroides*, the ileum-specific and feces-specific co-abundance relationships were involved in more OTUs from different taxa. Especially, significant negative correlations were identified between OTUs belonging to Lactobacillaceae and Clostridiaceae in the ileum of F6 pigs. Lactobacillaceae are abundant in the gut and can ferment carbohydrates into lactic acid which improves the gut environment (Chen et al., 2018). It has been reported that *Clostridiaceae* was a predominant taxonomy in the porcine ileum. The members in *Clostridiaceae* show the ability to metabolize plant-derived polysaccharide (Pollock et al., 2021). We inferred that the competition between Lactobacillaceae and Clostridiaceae for the utilization of carbohydrates might mediate the negative correlations between them. The colonization of bacterial species in Lactobacillaceae could fully exercise its mutual exclusivity with the members of Clostridiaceae. We found a large number of SCFA-producing microbes in the ileum microbiota of healthy pigs, and they showed significantly positive correlations with each other. The stable interactions among these microbes in the ileum suggested that the intestines of healthy experimental pigs had a stable capacity to produce and utilize SCFAs. Elevating the concentration of SCFAs in the ileum should further increase the concentration of SCFAs in serum and intestine, which benefits for keeping the gut barrier integrity (Diao et al., 2017). Different patterns of the gut location-specific co-abundance relationships were observed between F6 and F7 pig populations. This should be caused by differentiated genetic background of F6 and F7 pigs from the mosaic population and different harvested time of microbial samples (2006 vs. 2008).

The topological features of microbial co-abundance networks not only reveal spatial differentiation of the gut microbiota across intestinal regions, but also reflect the variations in microbial community stability, cooperation, and adaptation to host niches. Previous studies have demonstrated that similar network metrics (e.g., clustering coefficient, modularity, and centrality) could serve as valuable indicators for evaluating microbial functional interactions, ecological robustness, and potential targets for intervention (Huang et al., 2023). Therefore, our findings further support the use of network topology as a powerful framework for understanding the functional states of the gut microbial ecosystem.

In summary, with large scale of microbial samples from three gut locations of two pig populations, we analyzed the gut microbial compositions of healthy pigs and constructed the co-abundance networks in each of three gut locations. The findings suggested that the microbial composition was highly gut location-specific. Phylogenetic co-abundance network analysis showed that the co-abundance patterns of the gut microbiota were highly spatial heterogeneity and changed significantly along the intestinal niche. We characterized the topological characteristics of the co-abundance network in each gut location and identified stable and gut location-specific co-abundance relationships. These findings revealed the spatial ecological relationships of microbial communities among ileum, cecum, and feces. The results gave meaningful biological insights into the distribution of commensal bacteria and their interaction networks in different gut locations and provided basic knowledge for improving gut health and production performance in pigs by regulating gut microbiota targeting to specific gut locations.

## Data Availability

The original contributions presented in the study are publicly available. This data can be found here: https://ngdc.cncb.ac.cn/gsa/, accession numbers CRA006230 and CRA006239.

## Supplementary material

SUPPLEMENTARY FIGURE S1 Comparison of the microbial composition and diversity among three gut locations in F7 pigs. Comparison of the bacterial composition among ileum, cecum, and feces at the phylum **(A)** and genus level **(B)**. **(C)** Comparison of the alpha-diversity of microbial composition among ileum (*n* = 411), cecum (*n* = 651), and feces (*n* = 590). ACE, Chao1, observed species, Shannon, and Simpson indices were analyzed. * *p* < 0.05, ** *p* < 0.01, *** *p* < 0.001. **(D)** PCoA analysis of the microbial composition in ileum, cecum, and feces based on Bray-Curtis distance.

SUPPLEMENTARY FIGURE S2 Co-abundance relationships among OTUs detected in each of three gut locations of F7 pigs. **(A)** The number of co-abundance relationships identified in the ileum, cecum and feces of the F6 pig population (left) and the number of OTUs involved in these co-abundance relationships (right); **(B)** The changes in the numbers of co-abundance relationships identified in the ileum, cecum and feces of the F6 populations following the increase of correlation coefficients. The *X*-axis represents correlation coefficients, and the *Y*-axis indicated the number of OTUs with co-abundance relationships. **(C)** The number of co-abundance relationships shared among different gut locations following the increase of correlation coefficients in the F7 populations. The *X*-axis represents correlation coefficients, and the *Y*-axis indicates the number of OTU co-abundance relationships. **(D)** Evaluating the stability of co-abundance relationships between two gut locations in the F7 population. Each point represents a co-abundance relationship among OTUs. The horizontal and vertical axes represent the correlation coefficients of co-abundance relationships. Red dots indicate co-abundance relationships with no significant difference in effect size between two gut locations with *p* > 0.05 in Cochran's Q test. **(E)** The numbers of co-abundance relationships shared between two gut locations and among all three gut locations (boxplot) and the distribution of the correlation coefficients for 247 stable co-abundance relationships (pie chart). The values on the outside circle of the pie chart represent different correlation coefficients, and the values in brackets are the ratios of stable co-abundance relationships in 247 stable co-abundance relationships in each correlation coefficient interval.

SUPPLEMENTARY FIGURE S3 Modules of co-abundance networks constructed in the ileum, cecum and feces samples in the F7 population. The numbers of modules in the networks of ileum, cecum and feces were 6, 38, and 37, respectively. The correlations with coefficients >0.5 were used to constructed the co-abundance networks. Different colors meant different modules, the node size represents the degree of OTUs, red lines mean positive correlations and green lines indicate negative correlations. The thickness of the line between nodes shows the weight of the correlation coefficient between OTUs. **(A)** Ileum, **(B)** Cecum, and **(C)** feces.

SUPPLEMENTARY FIGURE S4 Gut location-specific co-abundance relationships in F7 pigs. **(A)** The proportion of gut location-specific co-abundance relationships identified in three gut locations of the F6 population. **(B)** The proportion of gut location-specific positive and negative relationships in the co-abundance networks of the ileum, cecum, and feces of the F6 population. **(C–E)** Co-abundance relationships specifically identified in the ileum **(C)**, cecum **(D)**, and feces **(E)** of F6 population at the taxonomic level. Each dot represents a microbial taxonomy. Each line represents a co-abundance relationship from the same or different taxa. The number of lines represents the number of co-abundance relationships identified in three gut locations.
