# Supplementary figures and images for: Topological and spatial heterogeneity of gut microbiota co-abundance networks in pigs revealed by using large-scale samples
Source: Front Microbiol. 2025 Jun 25;16:1578236. doi: 10.3389/fmicb.2025.1578236 (PMC12237903; doi:10.3389/fmicb.2025.1578236)

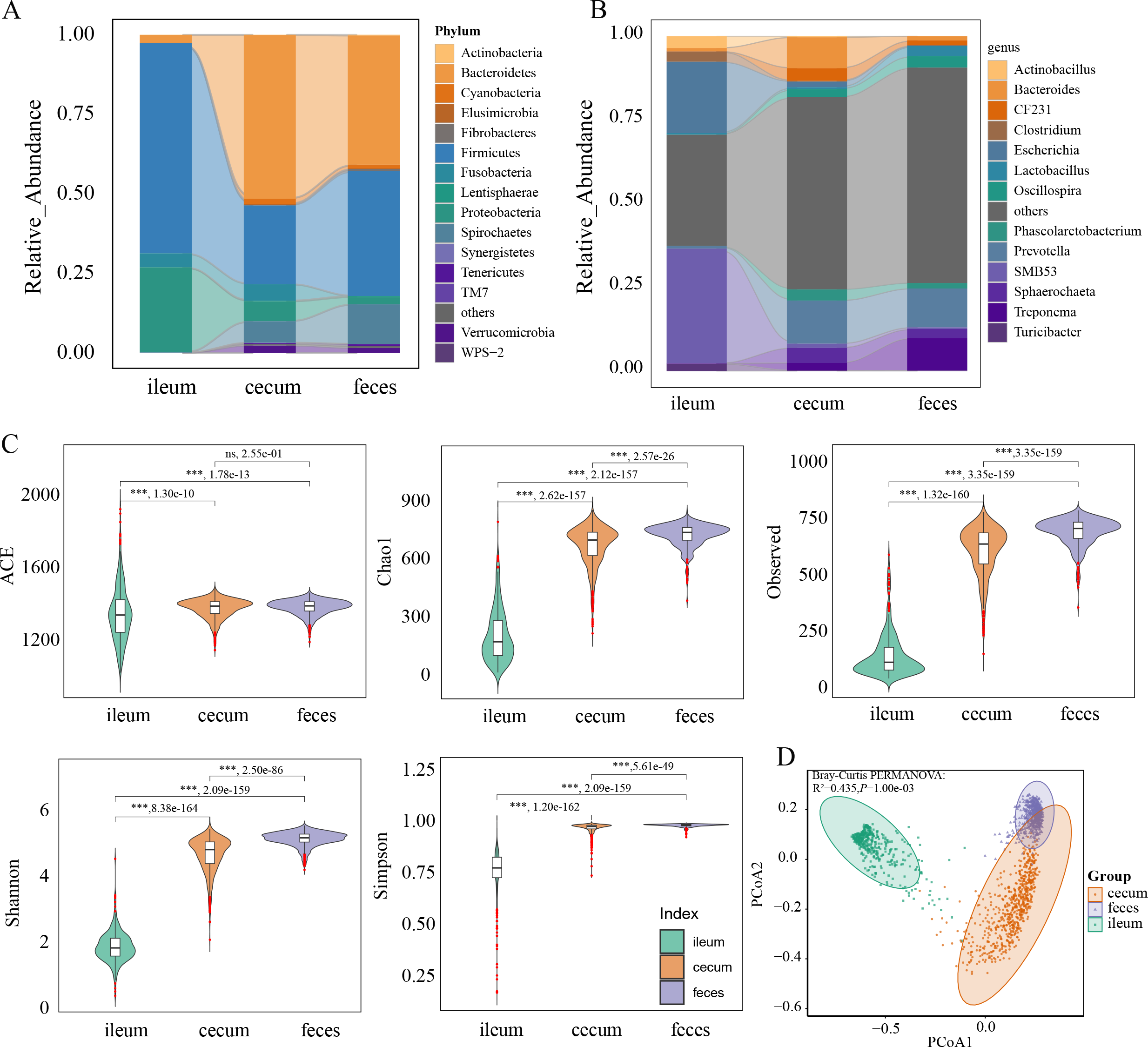

Supplement: SUPPLEMENTARY FIGURE S1 — Comparison of the microbial composition and diversity among three gut locations in F7 pigs. Comparison of the bacterial composition among ileum, cecum, and feces at the phylum (A) and genus level (B). (C) Comparison of the alpha-diversity of microbial composition among ileum (n = 411), cecum (n = 651), and feces (n = 590). ACE, Chao1, observed species, Shannon, and Simpson indices were analyzed. * p < 0.05, ** p < 0.01, *** p < 0.001. (D) PCoA analysis of the microbial composition in ileum, cecum, and feces based on Bray-Curtis distance. [file Image_1.tif]

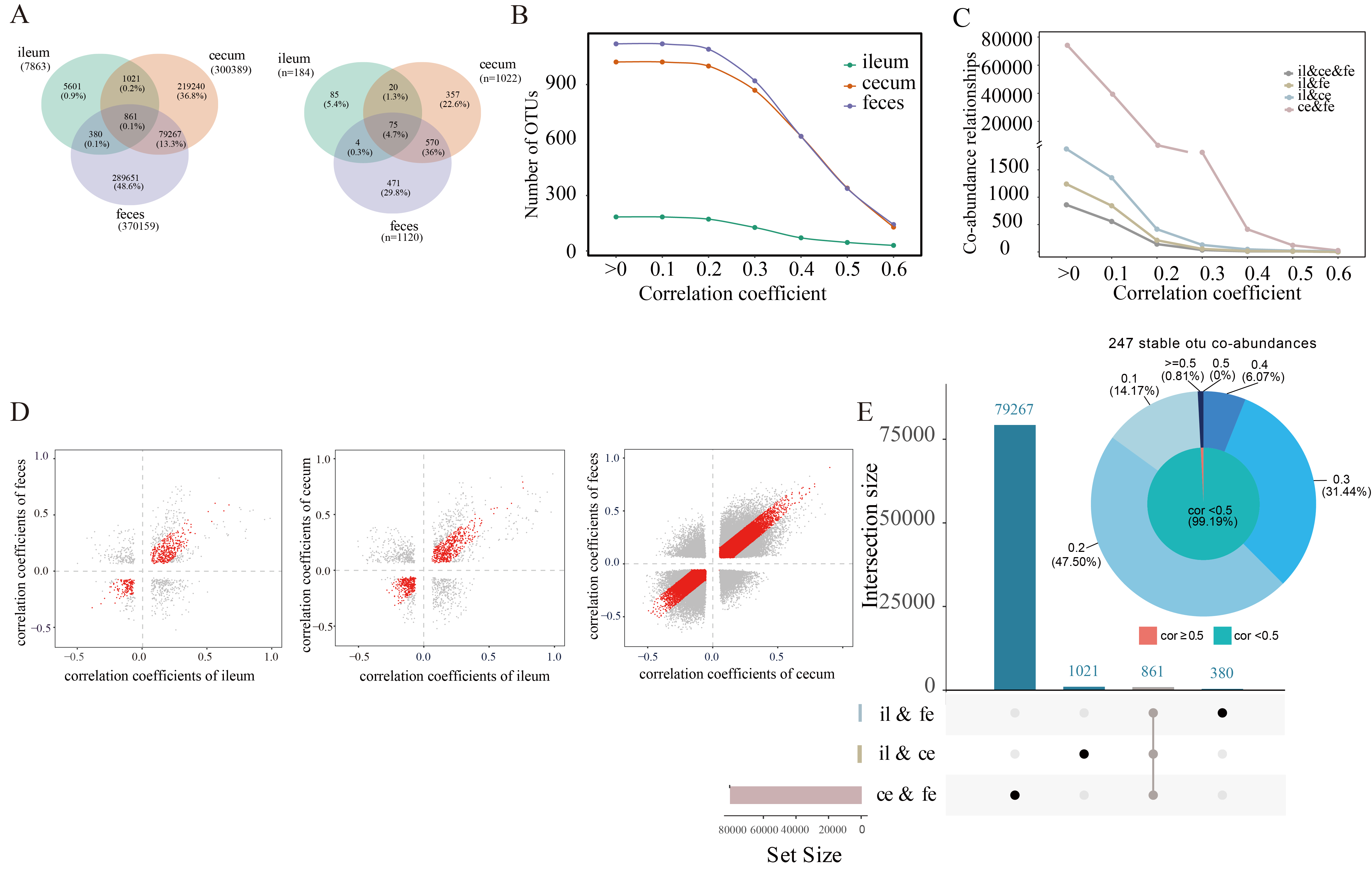

Supplement: SUPPLEMENTARY FIGURE S2 — Co-abundance relationships among OTUs detected in each of three gut locations of F7 pigs. (A) The number of co-abundance relationships identified in the ileum, cecum and feces of the F6 pig population (left) and the number of OTUs involved in these co-abundance relationships (right); (B) The changes in the numbers of co-abundance relationships identified in the ileum, cecum and feces of the F6 populations following the increase of correlation coefficients. The X-axis represents correlation coefficients, and the Y-axis indicated the number of OTUs with co-abundance relationships. (C) The number of co-abundance relationships shared among different gut locations following the increase of correlation coefficients in the F7 populations. The X-axis represents correlation coefficients, and the Y-axis indicates the number of OTU co-abundance relationships. (D) Evaluating the stability of co-abundance relationships between two gut locations in the F7 population. Each point represents a co-abundance relationship among OTUs. The horizontal and vertical axes represent the correlation coefficients of co-abundance relationships. Red dots indicate co-abundance relationships with no significant difference in effect size between two gut locations with p > 0.05 in Cochran's Q test. (E) The numbers of co-abundance relationships shared between two gut locations and among all three gut locations (boxplot) and the distribution of the correlation coefficients for 247 stable co-abundance relationships (pie chart). The values on the outside circle of the pie chart represent different correlation coefficients, and the values in brackets are the ratios of stable co-abundance relationships in 247 stable co-abundance relationships in each correlation coefficient interval. [file Image_2.tif]

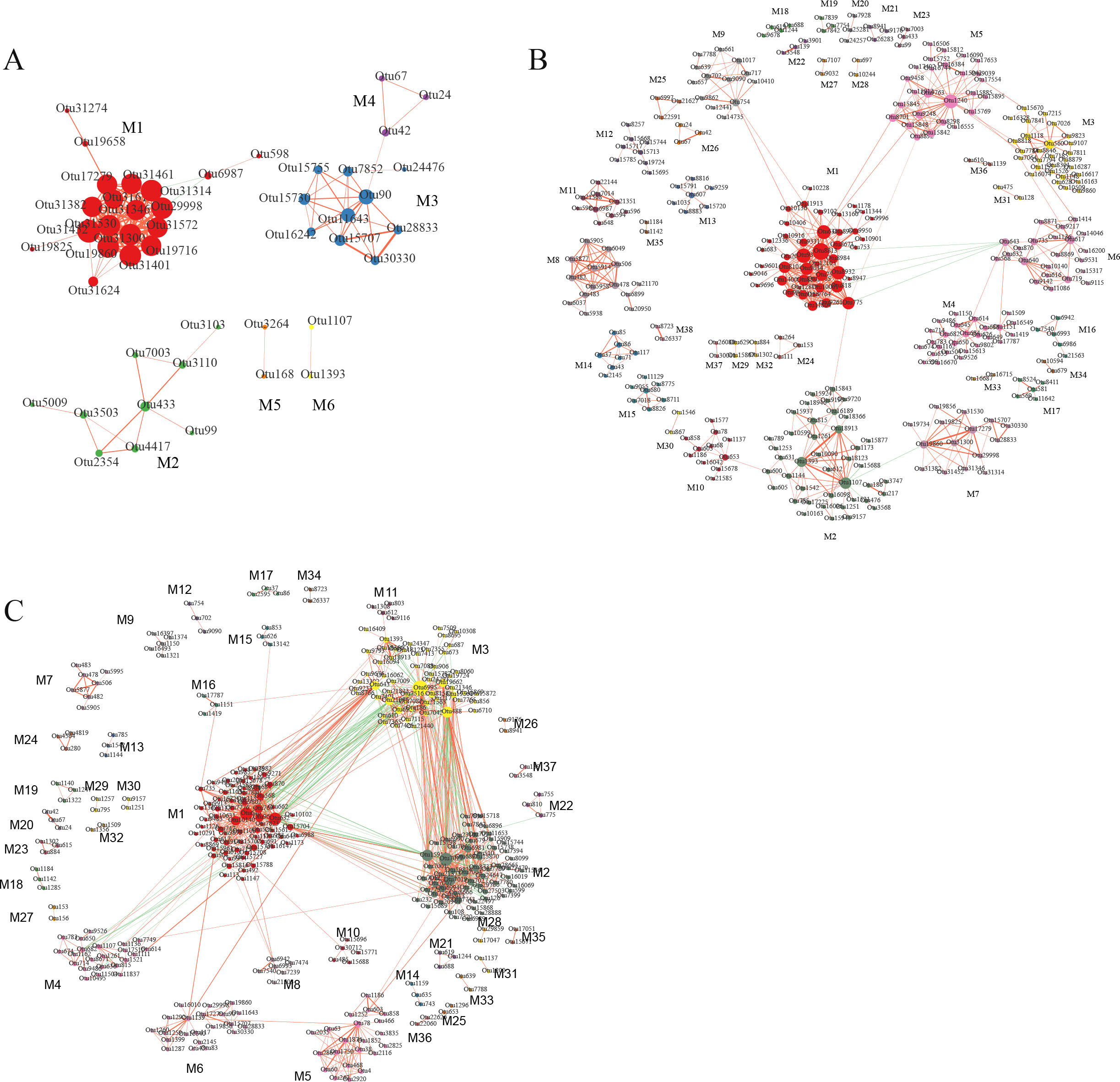

Supplement: SUPPLEMENTARY FIGURE S3 — Modules of co-abundance networks constructed in the ileum, cecum and feces samples in the F7 population. The numbers of modules in the networks of ileum, cecum and feces were 6, 38, and 37, respectively. The correlations with coefficients >0.5 were used to constructed the co-abundance networks. Different colors meant different modules, the node size represents the degree of OTUs, red lines mean positive correlations and green lines indicate negative correlations. The thickness of the line between nodes shows the weight of the correlation coefficient between OTUs. (A) Ileum, (B) Cecum, and (C) feces. [file Image_3.tif]

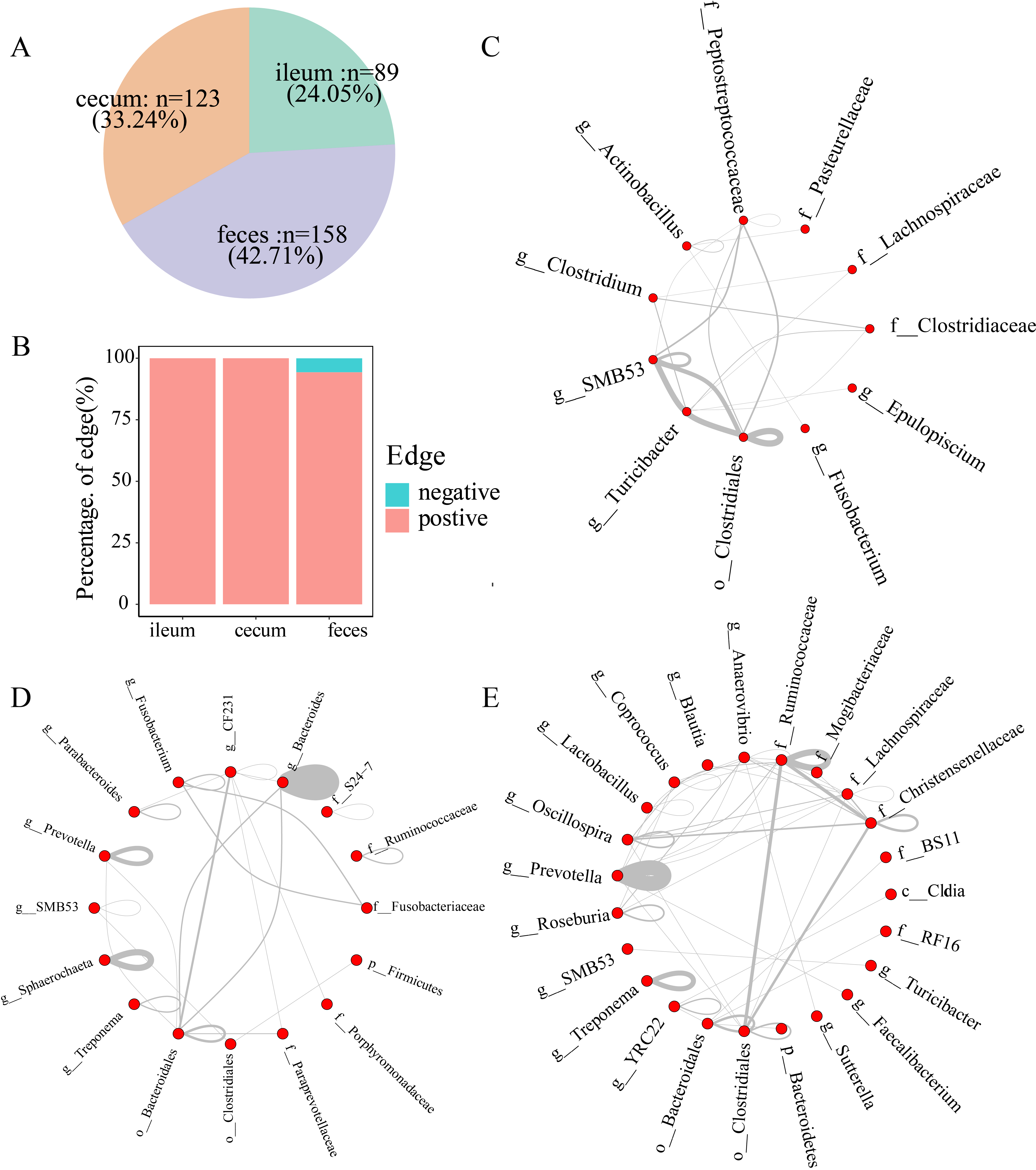

Supplement: SUPPLEMENTARY FIGURE S4 — Gut location-specific co-abundance relationships in F7 pigs. (A) The proportion of gut location-specific co-abundance relationships identified in three gut locations of the F6 population. (B) The proportion of gut location-specific positive and negative relationships in the co-abundance networks of the ileum, cecum, and feces of the F6 population. (C–E) Co-abundance relationships specifically identified in the ileum (C), cecum (D), and feces (E) of F6 population at the taxonomic level. Each dot represents a microbial taxonomy. Each line represents a co-abundance relationship from the same or different taxa. The number of lines represents the number of co-abundance relationships identified in three gut locations. [file Image_4.tif]
